# Transgender Health Medical Education Intervention and its Effects on Beliefs, Attitudes, Comfort, and Knowledge

**Published:** 2018-11-29

**Authors:** Joseph Cherabie, Kari Nilsen, Sarah Houssayni

**Affiliations:** 1 Department of Internal Medicine, University of Kansas School of Medicine-Wichita; 2 Department of Family and Community Medicine, University of Kansas School of Medicine-Wichita

**Keywords:** health services for transgendered persons, LGBT persons, healthcare disparities, medical education

## Abstract

**Introduction:**

Transgender health disparities have been well documented in the literature in recent years, as have the lack of transgender health issues in medical education programs across the country.

**Methods:**

A prospective study was conducted with an hour-long didactic lecture on transgender health being given to faculty, medical students, and residents at the University of Kansas School of Medicine-Wichita. The didactic lecture included educational information and presentations by transgender persons. A pre-intervention and two post-intervention survey was given to assess attitudes, comfort level, knowledge, and beliefs regarding the treatment of transgendered persons and associated health concerns. A second post-intervention survey was given at 90 days. The question of what attendees planned to do differently as a result of the intervention was asked.

**Results:**

The intervention provided a significant positive increase in attitudes, comfort levels, and knowledge with respect to transgender health issues between the pre- and post-intervention surveys, however, did not provide a significant positive increase in beliefs on transgender health issues. There was no significant change in attitude, comfort levels, knowledge, or beliefs from the post-survey after 90 days. Four categories of what attendees planned to do differently as a result of the intervention also were identified.

**Conclusions:**

A didactic lecture on transgender health issues can positively change attitudes, comfort levels, and knowledge on transgender health issues significantly with the changes sustaining after 90 days. Beliefs tend to be much harder to change.

## INTRODUCTION

The term “transgender” is used to describe individuals whose preferred gender identity and/or gender roles do not conform to their sex assigned at birth.^1^ Recent estimates showed that 1.4 million individuals in the United States identify as transgender, accounting for 0.6% of the population.^2^

Studies regarding transgender persons have increased in recent years and have shown a high prevalence of negative health outcomes including sexually transmitted diseases, mental health issues, and substance use disorders.^3^,^4^ There also has been an increase in the number of studies published on the topic of transgender health between the years of 2008 – 2018. The Institute of Medicine (IOM) published the first comprehensive report of its kind on lesbian, gay, bisexual, and transgender (LGBT) health and showed that transgender people experience stigma and discrimination from childhood to later adulthood.^5^ One report showed that 70% of transgender individuals experience discrimination, particularly in the healthcare setting.^6^ In a study on discrimination of delay of healthcare in transgender men and women, 30.8% of participants were found to delay or not seek needed health care due to discrimination. All of this has prompted organizations such as the IOM, Association of American Medical Colleges, American Medical Association, and the American Psychiatric Association to call for improved provider education on transgender issues to target these health disparities, starting from medical school to graduate medical education and onwards.

A recent survey of 176 medical schools in the United States and Canada showed the median reported time dedicated to LGBT-related content in the entire medical school curriculum was five hours.^7^ Furthermore, nine schools reported that no time was spent on LGBT-related content during preclinical years, and 44 schools reported that no time was spent during clinical years. Another survey of 464 residents and attending physicians showed that the majority of respondents did not discuss sexual orientation or gender identity with their patients, with 41% stating they did not discuss these topics with sexually active adults, citing a lack of training in dealing with these topics.^8^

In an assessment on the current state of transgender health care, Stroumsa wrote that “bias against transgender people takes an enormous toll on their health through direct harm, lack of appropriate care, and a hostile environment and through transgender people’s avoidance of the medical system as a result of discrimination and lack of respect”.^9^ She pointed out that the medical establishment has a duty to provide proper healthcare to transgender individuals and that this must be incorporated into medical curricula.

There have been many reports of interventions aimed at changing knowledge, attitudes, and beliefs of resident physicians and medical students. One intervention included a 90-minute workshop for psychiatry residents with pre-, post-, and 90-day follow-up surveys to “assess perceived empathy knowledge, comfort, interview skill, and motivation for future learning”.^10^ With this intervention, there was a statistically significant increase in perceived empathy, knowledge, comfort, and motivation for future learning in the short term. In another intervention, medical students attended a lecture and completed surveys assessing transgender health knowledge, attitudes, and skills, with follow-up surveys upon graduation.^11^ Participants showed significantly increased levels of competency compared to students who had not received the lecture, with higher average summary scores for overall self-reported knowledge, more positive attitudes, and skills. They also showed low baseline receipt of transgender education prior to entering residency.

In light of these reports and successes, an intervention was conducted with the aim of positively improving knowledge, attitudes, comfort, and beliefs in dealing with transgender health issues.

## METHODS

Residents and residency faculty, as well as medical students, attended an hour-long didactic lecture on transgender health. These didactic sessions included educational information about transgender health and appropriate medical treatment^4^, as well presentations from a male-to-female and a female-to-male transgender person regarding their transition and the medical care they received during that time in their life.

Participants received a pre-intervention survey before the session and an identical post-intervention survey at the end of the session to measure immediate change in beliefs (what they think about transgender patients), attitudes (how well they understand issues faced by transgender patients), comfort (how they feel treating transgender patients), and knowledge (what they know about medical care for transgender patients). Examples of questions regarding beliefs were “I think God made man and woman, anything else is abnormal” and “I think transgender people are sick”. Examples of questions regarding attitudes were “I understand the types of discrimination that transgender people face” and “I understand the difference between biologic sex, gender identity, and sexual orientation”. Examples of questions regarding comfort were “I feel comfortable using language that respects gender identity” and “I feel comfortable discussing options for gender confirming hormone therapy”. Examples of knowledge questions were “I know what resources about transgender health are available to me as a medical provider” and “I know ways to make a medical practice more transgender-friendly”.

The post-intervention survey was nearly the same as the pre-intervention survey, but included an open-ended question regarding what they planned to do differently in their practice as a result of the session. A 90-day post-intervention survey identical to the pre-intervention survey was given to participants who provided their email at the didactic lecture. A convenience sample of residents and faculty completed the survey. No identifiable information was collected and all participation was voluntary. The hosting Institutional Review Board approved the study as non-human subjects research.

### Statistical analysis

Responses were calculated for the four variables of beliefs, attitudes, comfort, and knowledge regarding transgender patients. Responses to survey questions were scored on a five-point Likert scale ranging from strongly disagree (1) to strongly agree (5). Paired samples t-tests were used to compare the population means and to assess for significant changes in each field between pre- and post-intervention surveys. Post-intervention data were compared to the 90-day post-intervention survey data. All data analyses were performed using SPSS version 24.0 (IBM, Armonk, NY) and Microsoft Excel^©^.

## RESULTS

**Figure 1:**
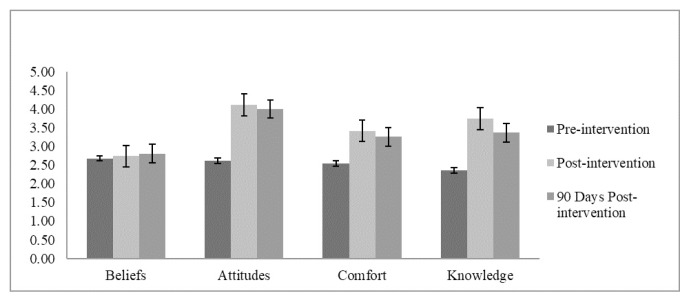
Beliefs, attitudes, comfort, and knowledge changes toward transgender patients from time one (pre-intervention), to time two (post-intervention), and time three (post 90-day intervention).

One hundred and sixty three individuals completed the pre-intervention survey. Of these, 53 were from family medicine (FM), 28 from internal medicine (IM), 23 from pediatrics, 46 from psychiatry, and 13 were medical students. Of the initial 163 participants, 115 (70.6%; 24 IM, 20 pediatrics, 35 psychiatry, and 13 medical students) participated in the post-intervention survey. The 90-day post-intervention survey was completed online by 18 individuals (11%; 12 IM, 6 medical students). Means and standard differences are shown in Table 1 for all four scales. Figure 1 shows a visual representation of the differences in the four scales for each time period.

### Beliefs

There was no significant change in mean difference between pre- and post-intervention survey responses for beliefs regarding transgender patients (t[272] = 1.05, p = 0.24, 95% CI −0.05 to 0.17), with mean responses of 2.68 (± 0.44) pre-intervention and 2.74 (± 0.50) post-intervention. There was also no significant change in beliefs between post-intervention and 90-day post-intervention surveys, with a mean score 90-day post-intervention survey being 2.81 (± 0.61) and a mean difference of 0.07 (t[130] = .52, p = 0.60, 95% CI −0.19 to 0.33).

### Attitudes

There was a significant change in the mean difference between pre and post-intervention survey responses for attitudes towards transgender patients (t[271] = 16.90, p < 0.0001, 95% CI 1.33 to 1.67), with the mean responses being 2.62 (± 0.81) pre-intervention and 4.12 (± 0.58) post-intervention. There was no significant change in positive attitudes when comparing the post-intervention and 90-day post-intervention survey with mean score 90-day post-intervention survey being 4.00 (± 0.61) and a mean difference of −0.12 (t[129] = −0.79, p = 0.43, 95% CI −0.42 to 0.18).

### Comfort

There was a significant change in the mean difference between pre and post-intervention survey responses for comfort in treating transgender patients (t[267] = 9.95, p < 0.0001, 95% CI 0.71 to 1.05), with the mean responses being 2.54 (± 0.81) pre-intervention and 3.42 (± 0.55) post-intervention. There was no significant change in comfort when comparing the post-intervention and 90-day post-intervention survey with mean score 90-day post-intervention survey being 3.26 (± 0.59) and a mean difference of −0.12 (t[126] = −1.11, p = 0.27, 95% CI −0.45 to 0.13).

### Knowledge

There was a significant change in the mean difference between pre- and post-intervention survey responses for knowledge regarding transgender patients (t[264] = 12.83, p < 0.0001, 95% CI 1.17 to 1.59), with the mean responses being 2.36 (± 0.90) pre-intervention and 3.74 (± 0.81) post-intervention. There was no significant change in knowledge when comparing the post-intervention and 90-day post-intervention survey with mean score 90-day post-intervention survey being 3.37 (± 0.78) and a mean difference of −0.12 (t[125] = −1.76, p = 0.08, 95% CI −0.79 to 0.05).

### Changes identified in post-intervention survey

Of the 115 attendees who completed a post-intervention survey, fifty-seven answered the open-ended question of “what do you plan to do differently in your practice as a result of this educational session?” Two members of the research team categorized the responses and came to a consensus of four categories: increased consideration for transgender patients, increased screening for gender dysphoria, continuing education into transgender medicine, and providing more treatment options for patients (α = 0.85). Any disagreements between the categories of quotes were broken by the third member of the research team. Some answers had multiple categories identified in one response for an overall number of 68 individual quotes in the four categories. Below are the categories and examples of the responses in each.

1. Increased consideration for transgender patients (n = 33, 49%)
  - “Integrating easy administrative changes in the office”
  - “Think about and ask carefully the questions and unconscious roles I assign”
2. Increased screening for gender dysphoria (n = 15, 22%)
  - “Be more aware of unique transgender screening needs”
  - “Be more thorough about screening for gender dysphoria”
3. Continuing education into transgender medicine (n = 11, 16%)
  - “Alter counseling and education to address medical issues specific to trans health”
  - “Do more reading/research on this topic”
4. Providing more treatment options for patients (n = 9, 13%)
  - “Learn more about possible treatments and labs”
  - “I plan to discuss options with patients”

## DISCUSSION

Within the study participants, the intervention showed a significant positive increase in attitudes, comfort, and knowledge towards transgender individuals between the pre- and post-intervention intervention surveys. The intervention, on the other hand, did not impact beliefs about transgender individuals. This may be due to already favorable beliefs about transgender individuals prior to the intervention. With respect to the 90-day post-intervention survey results, there were no significant changes, which is good, as this shows stable levels after 90 days. However, the 90-day post-intervention survey suffered from a low response rate of only 11%. Another limitation was that there were no post-intervention surveys with the family medicine audience. Also, individual responses were not able to be paired between the three administrations of the survey.

Of note, the lowest mean pre-intervention score was knowledge about transgender individuals, with the highest mean difference post-intervention, pointing to a possible deficiency within this program with respect to transgender health issues in the medical education curriculum. The fact that many people were going to make an effort to show more consideration for transgender patients, as well as increase screening and education, potentially means they had not thought about this issue previously.

This study was special in that it included participants from multiple residency programs as well as medical students, but this may have limited generalizability. While this increased the sample size of participants, it decreased our ability to focus in on deficiencies within particular residency programs as the sample sizes when each group were sampled individually were reduced. Questions regarding intent to treat transgendered patients also would be a helpful addition to the survey in the future to see if the intervention changed these intentions at all, as would repetition of the survey on a longitudinal basis to judge changes over a longer period of time. Utilizing larger groups from the different medical specialties in order to evaluate program differences would also be a useful change in future iterations of this intervention.

An area of future research is to look at individual programs and see if any program has greater deficiencies or benefits more from the intervention than others. Another possible area of interest would be to see if individual participants had any prior training on transgender health issues and if that increased their baseline knowledge, attitudes, comfort, and beliefs on the subject. Finally, if didactic lectures could be standardized and distributed to be included as part of the medical education curriculum, both at the medical school and residency levels in multiple programs, then results could be pooled and impact could be seen at each level with a larger sample size.

## CONCLUSION

Exposure to educational information has the potential to impact attitudes, comfort in treating, and knowledge regarding transgendered persons, even in small doses. The more that medical students and physicians learn about transgender health issues, the better care they will be able to provide for this growing population. This is an important first step in improving the healthcare provided to transgendered persons.

## Figures and Tables

**Table 1:** Beliefs, attitudes, comfort, and knowledge changes toward transgender patients.

	Pre-Intervention		Post-Intervention		90-day Post-Intervention	
	M	SD	M	SD	M	SD
Beliefs	2.68	0.44	2.74	0.50	2.81	0.61
Attitudes	2.62	0.81	4.12	0.58	4.00	0.80
Comfort	2.54	0.81	3.42	0.55	3.26	0.59
Knowledge	2.36	0.90	3.74	0.81	3.37	0.78
